# Efficacy and safety of Bailing Capsule for the treatment of adult primary nephrotic syndrome: a systematic review and meta-analysis of randomized controlled trials

**DOI:** 10.3389/fphar.2026.1798042

**Published:** 2026-04-24

**Authors:** Muyuan Guo, Tao Sun, Ruping Zhao, Qigang Guo, Yaoxian Wang, Kang Yang

**Affiliations:** 1 Treatment Center of Kidney Disease, the First Affiliated Hospital of Henan University of Chinese Medicine, ZhengZhou, China; 2 Collaborative Innovation Center of Prevention and Treatment of Major Diseases by Chinese and Western Medicine, Henan, ZhengZhou, China; 3 Henan University of Chinese Medicine, ZhengZhou, China

**Keywords:** bailing capsule, grade, meta, primary nephrotic syndrome (pns), randomized controlled trials (RCTs)

## Abstract

**Background:**

Bailing Capsule, a standardized fungal medicinal preparation recorded in the Chinese Pharmacopoeia (2020 Edition), is widely applied in the clinical management of various kidney diseases including adult primary nephrotic syndrome (PNS). However, the conclusions of existing clinical studies on its therapeutic effects for adult PNS remain inconsistent, lacking a comprehensive and quantitative evidence synthesis.

**Methods:**

A systematic review and meta-analysis of randomized controlled trials (RCTs) was conducted to evaluate the efficacy and safety of Bailing Capsule for adult PNS. Eight major databases were systematically searched from their inception to December 20, 2025, for relevant RCTs. Meta-analytical data processing was performed using Review Manager (RevMan) version 5.4, with no language restrictions applied to the literature search.

**Results:**

A total of 11 eligible RCTs involving 920 adult patients with PNS were included in this study. Compared with conventional therapy alone, Bailing Capsule (used as monotherapy or adjunctive therapy) significantly reduced levels of 24-hour urinary protein excretion (24hUpro), serum creatinine (Scr), blood urea nitrogen (BUN) and total cholesterol (TC), and markedly increased serum albumin (ALB), CD3^+^ T lymphocytes, CD4^+^ T lymphocytes and the CD4^+^/CD8^+^ ratio. Additionally, Bailing Capsule exhibited significant renoprotective effects, improved the overall clinical response rate, and presented a favorable safety profile with a low incidence of adverse events.

**Conclusion:**

Bailing Capsule may serve as a safe and effective adjunctive therapeutic option for adult patients with PNS, with positive effects on improving renal function indicators, regulating immune function and enhancing clinical efficacy. However, further high-quality, large-scale and multicenter RCTs are still required to validate the long-term efficacy and safety of Bailing Capsule for adult PNS.

## 1 Introduction

Adult primary nephrotic syndrome (PNS) is a group of glomerular disorders characterized by massive proteinuria, hypoalbuminemia, edema, and hyperlipidemia. Its pathogenesis is closely associated with injury to the glomerular filtration barrier and dysregulated immune and inflammatory responses (Kodner, 2016). Minimal change disease (MCD), primary focal segmental glomerulosclerosis (FSGS), and membranous nephropathy (MN) represent the major pathological subtypes of PNS (Hull and Goldsmith, 2008). The global prevalence of adult PNS has shown a steady increase in recent years, with epidemiological data indicating an incidence of approximately 4.30 cases per 100,000 person-years (Vestergaard et al., 2023). Inadequate disease control may lead to rapid progression to end-stage renal disease (ESRD), substantially increasing the risk of cardiovascular complications and mortality, thereby imposing a considerable medical and socioeconomic burden (Go et al., 2021).

Currently, the standard treatment for PNS primarily relies on glucocorticoids in combination with immunosuppressive agents (Wang and Greenbaum, 2019). However, long-term use of these therapies is frequently associated with a wide range of adverse effects, including infections, leukopenia, anemia, gastrointestinal intolerance, and metabolic disturbances, which markedly impair patients’ quality of life (Howman et al., 2013). Consequently, there is a growing interest in identifying safe and effective complementary or alternative therapeutic strategies to improve clinical outcomes in patients with PNS (Han et al., 2020).

Bailing Capsule is a standardized fungal medicinal preparation that is officially recorded in the *Chinese Pharmacopoeia* (National Pharmacopoeia Committee of the People’s Republic of China, 2020). Its sole active component is Fermented *Hirsutella sinensis* Mycelium Powder, which refers to the dried mycelium of *H. sinensis* Liu et al. (1989) (the anamorph of *Ophiocordyceps sinensis* (Berk.) G.H. Sung et al., family Ophiocordycipitaceae; strain CS-C-Q80) produced via industrial submerged liquid fermentation (Rivera et al., 2014). As a precious traditional Chinese botanical drug fungus with a long clinical history, *O. sinensis* is characterized by sweet flavor and neutral nature in TCM, with core effects of tonifying the kidney and replenishing the lung, and reinforcing vital qi. It has long been used for chronic consumptive disorders caused by kidney and lung vacuity, laying a traditional theoretical foundation for its modern application in renal diseases. *Ophiocordyceps sinensis* harbors a diverse array of bioactive metabolites, including cordycepic acid (mannitol), polysaccharides, and adenosine (Zhao et al., 2014). Accumulating pharmacological evidence suggests that *O. sinensis* exhibits immunomodulatory, anti-inflammatory, and antioxidant properties, as well as renoprotective effects and the ability to reduce proteinuria (Tang et al., 2025; Ng and Wang, 2005; Zhu et al., 1998). Bailing Capsule has therefore been widely applied in the treatment of kidney-related diseases, and an increasing number of clinical studies have reported its potential therapeutic benefits (Tao et al., 2024; Zhou et al., 2024; Sheng et al., 2020).

In recent years, several randomized controlled trials (RCTs) have investigated the efficacy of Bailing Capsule in combination with conventional therapy for adult patients with PNS; however, their findings remain inconsistent. To date, a comprehensive synthesis and quantitative evaluation of the available evidence are lacking. Therefore, the present systematic review and meta-analysis aimed to incorporate relevant RCTs to rigorously assess the efficacy and safety of Bailing Capsule in the treatment of adult PNS, thereby providing high-quality evidence to inform clinical decision-making.

## 2 Methods

This meta-analysis was conducted in accordance with the Preferred Reporting Items for Systematic Reviews and Meta-Analyses (PRISMA) 2020 statement (Page et al., 2021). The study was registered in the international prospective systematic review registration platform (PROSPERO) with the registration ID CRD420251273359.

This systematic review and meta-analysis was conducted and reported in strict compliance with the Controlled Vocabulary and Minimum Reporting Standards for Botanical Medicine Systematic Reviews and Meta-Analyses (ConPhyMP) guidelines (https://ga-online.org/best-practice/; DOI: 10.3389/fphar.2022.953205) (Heinrich et al., 2022). The completed ConPhyMP reporting checklists are provided as Supplementary Material, with non-applicable items clearly marked.

### 2.1 Search strategy

Two independent reviewers systematically searched eight major online databases—Embase, Web of Science, Cochrane Library, Sinomed, VIP Database, Wanfang Database, CNKI, and PubMed—covering the period from the inception of each database to December 2025. The search strategy involved a combination of MeSH terms and free-text keywords. Keywords included “Bailing Capsule,” “fermented *O. sinensis* mycelium,” “nephrotic syndrome,” and others (detailed search strategies for each database and results are provided in Supplementary Material S1). No language restrictions were applied during the search. Additionally, the reference lists of included studies and relevant reviews were manually checked to ensure that no potentially relevant studies were overlooked.

### 2.2 Study selection

Two reviewers (M.G. and T.S.) independently screened the identified studies based on predefined inclusion and exclusion criteria. Any disagreements were resolved through consultation with a third reviewer (R.Z.). The inclusion criteria were as follows: (a) original RCTs; (b) adult patients (≥18 years old) with PNS, diagnosed according to internationally recognized criteria; (c) the intervention involved Bailing Capsule (used alone or in combination with conventional therapy) in the experimental group, and placebo or conventional therapy in the control group; (d) studies reported at least one of the following outcome measures before and after treatment: efficacy rate, incidence of adverse events, 24-h urinary protein quantification (24hUpro), plasma albumin (ALB), serum creatinine (Scr), total cholesterol (TC), CD3^+^ T lymphocytes (CD3^+^), CD4^+^ T lymphocytes (CD4^+^), CD4^+^/CD8^+^ ratio (CD4^+^/CD8^+^), and blood urea nitrogen (BUN). The exclusion criteria were: (a) studies involving children, pregnant, or breastfeeding women; (b) interventions involving Bailing Capsule combined with other traditional Chinese medicines or supplements; (c) study types such as case reports, reviews, conference abstracts, commentaries, letters to editors, or animal studies; (d) studies with incomplete data or inability to extract valid outcome measures; (e) studies not focusing on adult PNS.

### 2.3 Data extraction

Two independent reviewers (M.G. and T.S.) used a structured data extraction form to collect relevant information from eligible studies. Any discrepancies in data extraction were resolved by a third reviewer (R.Z.). The extracted data included: (a) basic study information: first author, year of publication, country, and study design type; (b) baseline patient characteristics: sample size, age, gender distribution, disease duration, etc.,; (c) intervention details: Bailing Capsule dosage, treatment duration, and control group interventions; (d) outcome measures: efficacy rate, incidence of adverse events, 24hUpro, ALB, Scr, TC, CD3^+^, CD4^+^, CD4^+^/CD8^+^, and BUN, along with the pre- and post-treatment means and standard deviations (SD).

### 2.4 Risk of bias assessment

Two independent reviewers (Q.G. and M.G.) assessed the risk of bias for the included studies using the Cochrane Risk of Bias 2.0 (ROB 2.0) (Sterne et al., 2019). The evaluation dimensions included bias arising from the randomization process, deviations from the intended interventions, missing data, measurement of outcomes, and selective reporting. Each reviewer categorized the risk of bias in each domain as “low risk,” “high risk,” or “some concerns.” Any disagreements were resolved through discussion or arbitration by a third reviewer (T.S.).

### 2.5 Statistical analysis

Meta-analysis was performed using Review Manager (RevMan) version 5.4. For continuous outcomes such as 24hUpro, ALB, Scr, TC, CD3^+^, CD4^+^, CD4^+^/CD8^+^, and BUN, the mean change and SD before and after treatment were used for quantitative analysis. If the SD of the mean change was not reported, the following formula was applied (Higgins et al., 2024): SD^2^_change_ = (SD^2^_pre-intervention_ + SD^2^_after-intervention_)-(2R × SD_pre-intervention_ × SD_after-intervention_) where the correlation coefficient (R) was set to 0.5. If the study reported the standard error (SE) instead of SD, the SD was calculated using the formula SD = SE × √N (Hozo et al., 2005). The pooled effect size was expressed as the weighted mean difference (WMD) with its 95% confidence interval (CI). For dichotomous outcomes (e.g., incidence of adverse events), risk ratios (RR) and their 95% CI were used. A P-value of <0.05 was considered statistically significant.

The heterogeneity among studies was assessed using the Higgins I^2^ statistic. If I^2^ ≥ 50% and P < 0.05, moderate to high heterogeneity was considered, and a random-effects model was used to pool effect sizes. If I^2^ < 50% and P ≥ 0.05, low heterogeneity was assumed, and a fixed-effects model was applied. Subgroup analyses based on the use of Bailing Capsule in combination with immunosuppressive agents were conducted to explore potential sources of heterogeneity. Funnel plots were generated for outcome measures with at least nine included studies to visually inspect for publication bias (Egger et al., 1997), and Egger’s test was employed to quantify publication bias. Sensitivity analysis was performed by sequentially excluding individual studies to test the robustness of the pooled results.

### 2.6 Quality of evidence assessment

The quality of the evidence for each meta-analysis result was assessed and graded using the online tool GRADEpro (https://www.gradepro.org/) (Guyatt et al., 2008). The evaluation covered five domains: risk of bias, inconsistency, indirectness, imprecision, and publication bias. The GRADE rating was independently performed by two reviewers (Q.G. and M.G.), and any disagreements were resolved through discussion or third-party arbitration (K.Y.).

### 2.7 Standardization guidelines for Bailing Capsule

Bailing Capsule is a standardized commercial proprietary Chinese medicine officially listed in the *Chinese Pharmacopoeia* (2020 Edition). It is manufactured from the fermented mycelium of *H. sinensis* (anamorph: *H. sinensis*, family Ophiocordycipitaceae) via a uniform industrial submerged fermentation process, encompassing standard strain activation, seed culture, liquid submerged aerated fermentation (22 °C–26 °C, pH 6.0–7.0, 7–10 days), harvest by centrifugation/filtration, washing, vacuum drying, milling, and final encapsulation. The formulation contains fermented *H. sinensis* mycelium powder as the sole active raw material and starch as the inactive excipient, with strict quality control based on three marker metabolites: adenosine, mannitol, and total amino acids (Table 1).

**TABLE 1:** Bailing capsule drug information.

Item	Pharmacopoeial standard details
Source strain	Fermented *Hirsutella sinensis* mycelium powder (CS-C-Q80); source: *Hirsutella sinensis* Liu, Guo, Yu-et Zeng (1989)
Core raw material	Fermented *Hirsutella sinensis* mycelium powder
Preparation method	200 g or 500 g of fermented *Hirsutella sinensis* mycelium powder is filled into 1,000 capsules
Description	Hard capsules, containing gray to grayish-yellow powder; slight fishy odor, slightly salty taste
Identification	1. TLC identification (using ergosterol as reference); 2. HPLC identification (6 main chromatographic peaks); 3. Total amino acid identification (tyrosine, lysine, histidine, arginine)
Assay – Mannitol	≥14 mg per capsule (0.2 g dosage form); ≥35 mg per capsule (0.5 g dosage form)
Assay – Adenosine	≥0.16 mg per capsule (0.2 g dosage form); ≥0.40 mg per capsule (0.5 g dosage form)
Assay – Total amino acids	≥60 mg per capsule (0.2 g dosage form); ≥150 mg per capsule (0.5 g dosage form)
Specifications	(1) 0.2 g per capsule; (2) 0.5 g per capsule
Excipient	Starch (only for capsule formulation, inactive)
Quality inspection	Complies with all general requirements for capsules (general Principle 0103)

All Bailing Capsules administered in the 11 included RCTs were authentic commercial products manufactured by Hangzhou Zhongmei Huadong Pharmaceutical Co., Ltd. (including Hangzhou Zhongmei Huadong Pharmaceutical Jiangdong Co., Ltd.), with the national medicine approval number Z10910036. This product is available as hard capsules in two commercial specifications (0.2 g and 0.5 g per capsule), containing gray to grayish-yellow powder with a slight fishy odor and mildly salty taste. Given the consistent raw materials, fermentation processes, and pharmacopoeial quality standards applied to all study preparations, the interventions were fully comparable across trials.

Regarding product traceability, all included studies confirmed the use of compliant products from the above manufacturer; however, specific production batch numbers, manufacturing dates, and expiration dates were not reported in the original publications. All investigational products were clinically sourced commercial drugs in full compliance with pharmaceutical Good Manufacturing Practice (GMP) and national drug traceability regulations, ensuring reliable product quality and safety.

## 3 Results

### 3.1 Study selection

**FIGURE 1:**
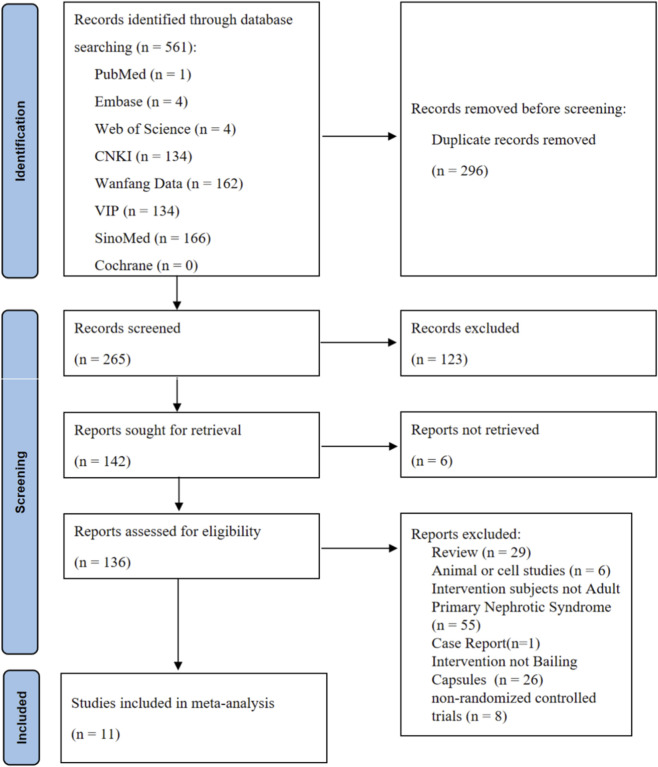
Flowchart of the eligible literature search process.

A total of 561 relevant studies were identified during the initial search (Figure 1). After removing 296 duplicate records, 265 studies remained for title and abstract screening. Of these, 123 studies were excluded for not meeting the inclusion criteria, and 142 studies proceeded to full-text review. During the full-text review process, 6 articles were excluded due to the inability to obtain the full text, and 125 studies were excluded for the following reasons: review articles (n = 29), animal or cell experiments (n = 6), case reports (n = 1), interventions not meeting the requirements (n = 26), participants not meeting the criteria (n = 55), and non-randomized controlled trial designs (n = 8). Ultimately, 11 eligible RCTs were included in this meta-analysis. No additional eligible studies were identified during the literature review phase.

### 3.2 Study characteristics

Table 2 summarizes the key characteristics of the included studies. All 11 studies were published between 2005 and 2022 and were conducted in China (Yue et al., 2006; Cao, 2009; Li, 2016; Xu, 2016; Chen and Wu, 2017; Feng, 2019; Wang, 2019; Chen et al., 2021; Cheng et al., 2021; Zhang, 2022; Chu et al., 2022). In terms of study design, all 11 studies were RCTs.

**TABLE 2:** Study characteristics.

Author and reference	Sample size		Age (years)		Interventions		Treatment course	Outcomes
​	T	C	T	C	T	C	​	​
Li (2016)	34	34	44.93 ± 8.21	43.58 ± 7.54	Conventional Tx + Atorva + BC	Conventional Tx + Atorva	Not specified	① Total effective rate; ② Renal function indicators (24hPr, BUN, TC, PT); ③ Adverse reactions
Yue et al. (2006)	28	26	60.5 ± 3.21	59.2 ± 4.15	Conventional Tx + BC	Conventional Tx (Dipyridamole, Lotensin, Hormone, etc.)	12 weeks	① Immunological indicators (IgG, IgM, IgA, CD3^+^, CD4^+^, CD4^+^/CD8^+^); ② Renal function indicators (BUN, Scr, 24 h urinary protein); ③ UNAG
Wang (2019)	45	45	49.25 ± 9.37	50.31 ± 9.05	Conventional Tx + BC	Conventional Tx (pred, Diuresis, anti-infection, etc.)	8 weeks	① Total effective rate; ② Renal function indicators (BUN, Scr, 24 h urinary protein, ALB); ③ 11-DH-TXB2, HMGB-1
Chen and Wu (2017)	30	30	51.63 ± 11.84	50.72 ± 11.26	Conventional Tx (pred, CTX, etc.) + BC	Conventional Tx (pred, CTX, etc.)	6 months	① Total effective rate; ② T lymphocyte subsets (CD3^+^, CD4^+^, CD8^+^, CD4^+^/CD8^+^); ③ Inflammatory indicators (hs-CRP, TNF-α)
Xu (2016)	24	24	51.72 ± 3.25	51.70 ± 3.24	Benazepril + BC	Benazepril	12 months	① Total effective rate; ② Plasma albumin level
Feng (2019)	50	50	30.8 ± 5.4	31.4 ± 5.3	Pred acetate + BC	Pred acetate	3 months	① Total effective rate; ② ALB, blood lipid indicators (TG, TC, LDL, HDL); ③ Renal function indicators (BUN, Scr, 24hTUP); ④ Inflammatory factors (TNF-α, IL-1β, IL-18); ⑤ Adverse reactions
Chen et al. (2021)	42	42	50.52 ± 9.83	51.43 ± 9.81	CsA + BC	CsA	6 months	① Total effective rate; ② Coagulation function (FIB, PT, APTT); ③ T lymphocyte subsets (CD3^+^, CD4^+^, CD8^+^, CD4^+^/CD8^+^); ④ Renal function indicators (BUN, Scr, ALB, 24 h urinary protein); ⑤ Adverse reactions
Chu et al. (2022)	42	42	48.92 ± 5.33	48.78 ± 5.29	Low-dose CsA + BC	CsA	6 months	① Total effective rate; ② Renal function indicators (ALB, 24hUTP, Scr, BUN, GFR); ③ Renal injury markers (KIM-1, RBP, NAG); ④ Immunological indicators (CD3^+^, CD4^+^, CD8^+^, CD4^+^/CD8^+^); ⑤ Adverse reactions
Cao (2009)	34	36	29.5 (19–54)	29.5 (19–54)	Pred + LMWH + BC	Pred	8 weeks	① Biochemical indicators (24hUpro, ALB, TC, BUN, Scr, PT, Plt); ② Total effective rate
Zhang (2022)	60	60	48.04 ± 9.37	47.92 ± 9.34	CTX + BC	CTX	8 weeks	① Total effective rate; ② Renal function indicators (24hPro, ALB, BUN, Scr); ③ Blood lipid indicators (TG, TC, HDL, LDL, ApoA1, ApoB)
Cheng et al. (2021)	52	52	42.19 ± 5.28	43.50 ± 5.41	Low-dose United Kingdom + BC	Low-dose United Kingdom	2 months	① Total effective rate; ② Renal function indicators (Scr, BUN, ALB); ③ Serum indicators (β2-MG, LPa, suPAR); ④ Adverse reactions

A total of 920 adult patients with PNS were included in this study, with 441 patients in the experimental group and 441 patients in the control group. The sample size of the included studies ranged from 48 participants (Xu, 2016) to 120 participants (Zhang, 2022). The overall weighted average age of the participants was approximately 45.7 years, including both male and female patients, with disease durations ranging from 1 month to 8 years.

In all included studies, the intervention in the experimental group was Bailing Capsule (either used alone or in combination with conventional therapy), while the control group received conventional therapy (including glucocorticoids, immunosuppressive agents, etc.) or a placebo. The daily dose of Bailing Capsule ranged from 1.8 to 9 g, with treatment durations ranging from 2 to 12 months. Specifically, four studies administered the intervention for 6 months (Chen et al., 2021; Chen and Wu, 2017; Zhang, 2022; Chu et al., 2022), two studies for 2 months (Cao, 2009; Cheng et al., 2021), two studies for 3 months (Feng, 2019; Yue et al., 2006), one study for 12 months (Xu, 2016), one study for 4 months (Wang, 2019), and one study did not specify the exact treatment duration (Li, 2016).

### 3.3 Risk of bias assessment

**FIGURE 2:**
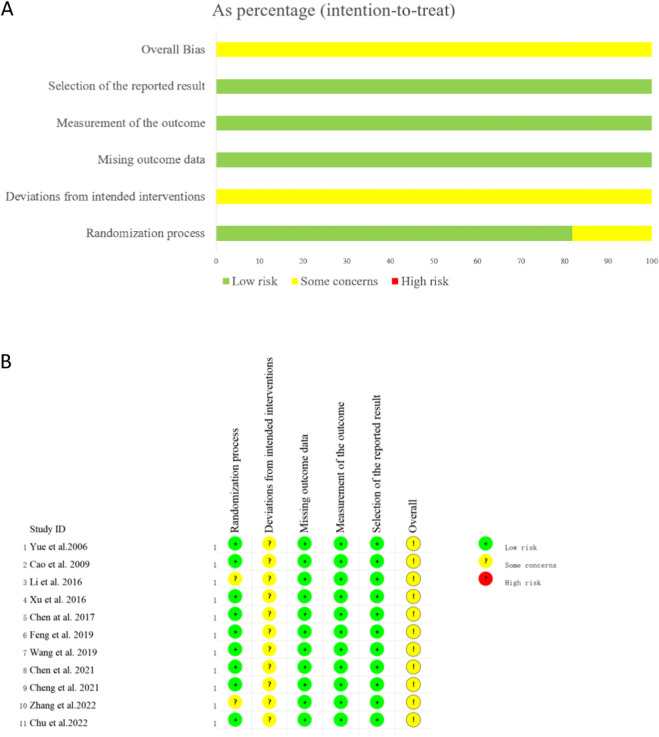
Risk of bias assessment of included RCTs (intention-to-treat population). **(A)** Proportion of studies with different bias risks per domain. **(B)** Bias risk judgment for each included study.

The overall quality of the included studies was relatively poor (Figure 2). The specific results of the risk of bias assessment are as follows: Two studies were assessed as having some concerns regarding the randomization process due to the unclear randomization methods (Li, 2016; Zhang, 2022). All 11 studies did not mention the implementation of blinding and were therefore judged to have some concerns in this regard (Yue et al., 2006; Cao, 2009; Li, 2016; Xu, 2016; Chen and Wu, 2017; Feng, 2019; Wang, 2019; Chen et al., 2021; Cheng et al., 2021; Zhang, 2022; Chu et al., 2022). For all studies, the risk of bias related to outcome data missingness, outcome measurement, and selective reporting was judged to be low. Overall, the risk of bias across all included studies was assessed as having some concerns.

### 3.4 Meta-analysis results

#### 3.4.1 Efficacy rate

**FIGURE 3:**
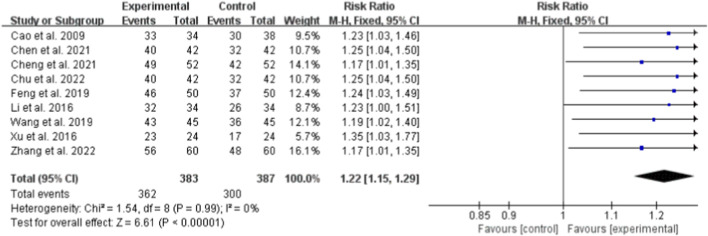
Forest plot of the effective rate: bailing capsule vs. conventional treatment group.

Nine studies (totaling 683 participants) assessed the effect of Bailing Capsule on clinical efficacy (Cao, 2009; Chen et al., 2021; Cheng et al., 2021; Chu et al., 2022; Feng, 2019; Li, 2016; Wang, 2019; Xu, 2016; Zhang, 2022). The meta-analysis demonstrated that the clinical efficacy rate in the Bailing Capsule intervention group was significantly higher than that in the control group (RR: 1.22; 95% CI: 1.15, 1.29; p < 0.00001). No significant heterogeneity was observed across the studies (I^2^ = 0%, p = 0.99; Chi^2^ = 5.64, df = 8) (Figure 3). Sensitivity analysis showed that the core trend of the pooled effect size remained unchanged after sequentially excluding any individual study, confirming the robustness of the results (Supplementary Figure S1. See Supplementary Matrial S2).

#### 3.4.2 24hUpro

**FIGURE 4:**
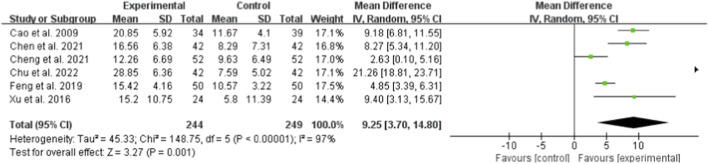
Forest plot of 24hUpro: bailing capsule vs. conventional treatment group.

Four studies (totaling 340 participants) assessed the impact of Bailing Capsule on 24hUpro (Cao, 2009; Chen et al., 2021; Chu et al., 2022; Feng, 2019). The meta-analysis revealed that Bailing Capsule significantly reduced 24hUpro after treatment (MD: −0.81 g/d; 95% CI: −1.17, −0.45; p < 0.001). High heterogeneity was observed across the studies (I^2^ = 71%, p = 0.02; Tau^2^ = 0.09, Chi^2^ = 10.38, df = 3) (Figure 4). Sensitivity analysis indicated that the core trend of the pooled effect size remained unchanged after sequentially excluding any individual study (the estimated values remained within the original 95% CI), and statistical significance was consistently maintained (p < 0.05 for all exclusions), confirming the robustness of the results (Supplementary Figure S2. See Supplementary Matrial S2).

#### 3.4.3 ALB

**FIGURE 5:**
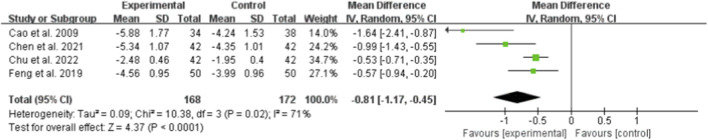
Forest plot of ALB: bailing capsule vs. conventional treatment group.

Six studies (totaling 249 participants) evaluated the effect of Bailing Capsule on ALB levels (Cao, 2009; Chen et al., 2021; Cheng et al., 2021; Feng, 2019; Chu et al., 2022; Xu, 2016). The meta-analysis indicated that Bailing Capsule significantly increased ALB levels after treatment (MD: 9.25 g/L; 95% CI: 3.70, 14.80; p = 0.001). A very high level of heterogeneity was observed across the studies (I^2^ = 97%, p < 0.00001; Tau^2^ = 45.33, Chi^2^ = 148.44, df = 5) (Figure 5). Sensitivity analysis showed that the core trend of the pooled effect size remained unchanged after sequentially excluding any individual study (the estimated values remained within the original 95% CI), and statistical significance was consistently maintained (p < 0.05 for all exclusions), confirming the robustness of the results (Supplementary Figure S3. See Supplementary Matrial S2).

#### 3.4.4 Scr

**FIGURE 6:**
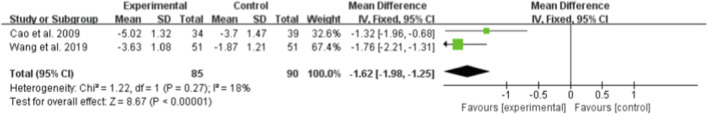
Forest plot of Scr: bailing capsule vs. conventional treatment group.

Six studies (totaling 201 participants) assessed the effect of Bailing Capsule on Scr levels (Cao, 2009; Feng, 2019; Chen et al., 2021; Wang, 2019; Yue et al., 2006; Cheng et al., 2021). The meta-analysis showed that Bailing Capsule significantly reduced Scr levels after treatment (MD: −10.98; 95% CI: −17.30, −4.66; p = 0.0007). Extremely high heterogeneity was observed across the studies (I^2^ = 99%, p < 0.00001; Tau^2^ = 41.78, Chi^2^ = 36.21, df = 4) (Figure 6). Sensitivity analysis demonstrated that the core trend of the pooled effect size remained unchanged after sequentially excluding any individual study (the estimated values remained within the original 95% CI), and statistical significance was consistently maintained (p < 0.05 for all exclusions), confirming the robustness of the results (Supplementary Figure S4. See Supplementary Matrial S2).

#### 3.4.5 TC

**FIGURE 7:**
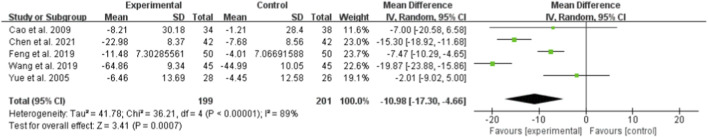
Forest plot of TC: bailing capsule vs. conventional treatment group.

Two studies (totaling 175 participants) assessed the effect of Bailing Capsule on TC levels (Cao, 2009; Wang, 2019). The meta-analysis showed that Bailing Capsule significantly reduced TC levels after treatment (MD: −1.62; 95% CI: −1.98, −1.25; p < 0.00001). Low heterogeneity was observed across the studies (I^2^ = 18%, p = 0.27; Chi^2^ = 1.22, df = 1) (Figure 7).

#### 3.4.6 T lymphocytes

**FIGURE 8:**
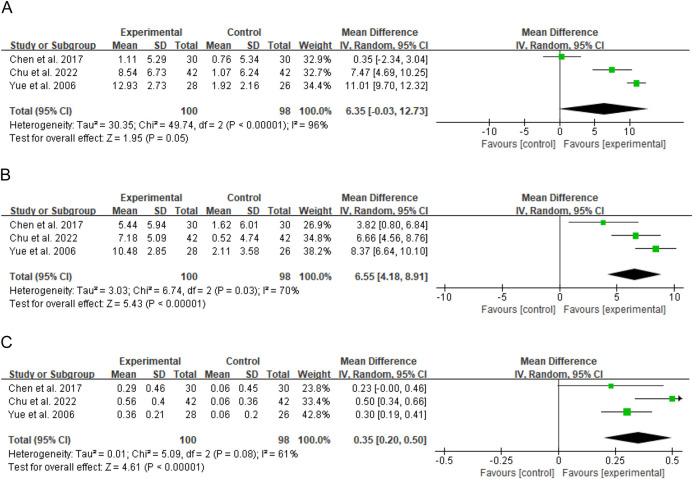
Forest plots of immune indicators: Bailing capsule vs. control group. **(A)** CD3^+^; **(B)** CD4^+^; **(C)** CD4^+^/CD8^+^.

Three studies (totaling 98 participants) evaluated the effect of Bailing Capsule on CD3^+^, CD4^+^, and CD4^+^/CD8^+^ (Chen and Wu, 2017; Chu et al., 2022; Yue et al., 2006). The meta-analysis showed that Bailing Capsule significantly increased CD3^+^ levels (MD: 6.35; 95% CI: 0.03, 12.73; p = 0.05), with extremely high heterogeneity observed (I^2^ = 96%, p < 0.00001; Tau^2^ = 30.35, Chi^2^ = 49.74, df = 2) (Figure 8a). Bailing Capsule also significantly increased CD4^+^ levels (MD: 6.55; 95% CI: 4.18, 8.91; p < 0.00001), with high heterogeneity across the studies (I^2^ = 70%, p = 0.03; Tau^2^ = 3.03, Chi^2^ = 6.74, df = 2) (Figure 8b). Furthermore, Bailing Capsule significantly increased the CD4^+^/CD8^+^ (MD: 0.35; 95% CI: 0.20, 0.50; p < 0.00001), with moderate heterogeneity (I^2^ = 61%, p = 0.08; Chi^2^ = 5.09, df = 2) (Figure 8c). Sensitivity analysis showed that the core trend of the pooled effect size remained consistent after sequentially excluding any individual study, indicating that the results were reasonably stable (Supplementary Figure S5. See Supplementary Matrial S2).

#### 3.4.7 BUN

**FIGURE 9:**
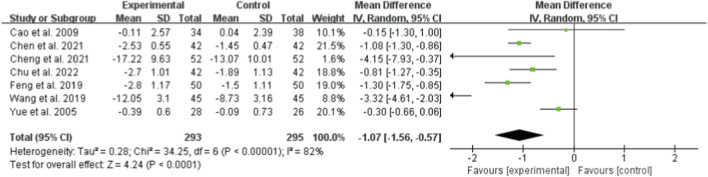
Forest plot of BUN: bailing capsule vs. conventional treatment group.

Seven studies (totaling 293 participants) assessed the effect of Bailing Capsule on BUN levels (Cao, 2009; Chen et al., 2021; Cheng et al., 2021; Chu et al., 2022; Feng, 2019; Wang, 2019; Yue et al., 2006). The meta-analysis revealed that Bailing Capsule significantly reduced BUN levels after treatment (MD: −1.07; 95% CI: −1.56, −0.57; p < 0.0001). High heterogeneity was observed across the studies (I^2^ = 82%, p < 0.00001; Tau^2^ = 0.28, Chi^2^ = 34.53, df = 6) (Figure 9). Sensitivity analysis showed that the core trend of the pooled effect size remained consistent after sequentially excluding any individual study, confirming the robustness of the results (Supplementary Figure S6. See Supplementary Matrial S2).

#### 3.4.8 Safety

**FIGURE 10:**
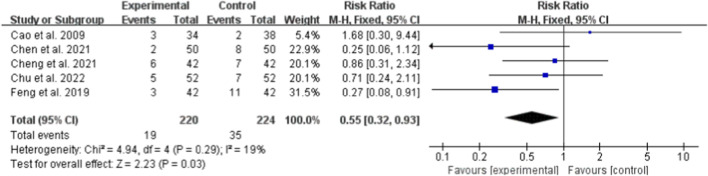
Forest plot of Safety: bailing capsule vs. conventional treatment group.

Five studies (totaling 444 participants) assessed the safety of Bailing Capsule (Cao, 2009; Chen et al., 2021; Cheng et al., 2021; Chu et al., 2022; Feng, 2019). The meta-analysis showed that the incidence of adverse events in the Bailing Capsule intervention group was significantly lower than in the control group (RR: 0.55; 95% CI: 0.32, 0.93; p = 0.03). Low heterogeneity was observed across the studies (I^2^ = 19%, p = 0.29; Chi^2^ = 4.94, df = 4) (Figure 10). Sensitivity analysis indicated that the core trend of the pooled effect size remained unchanged after sequentially excluding any individual study, confirming the robustness of the results (Supplementary Figure S7. See Supplementary Matrial S2).

#### 3.4.9 Subgroup analysis

To investigate the sources of heterogeneity in the core outcome measures, this study conducted subgroup analyses based on two dimensions: the use of concomitant immunosuppressants, and the dosage and duration of treatment with Bailing Capsule. Given the high collinearity between dosage and treatment duration in the included studies (all low-dose studies had short treatment durations, and all high-dose studies had long treatment durations), these two factors were combined into “low-dose/short-duration” and “high-dose/long-duration” subgroups for analysis.

##### 3.4.9.1 Subgroup analysis of 24Upro

**FIGURE 11:**
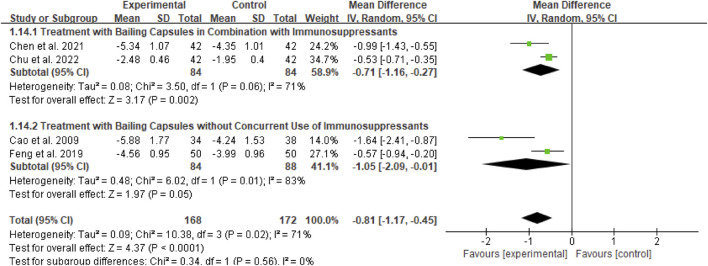
Subgroup analysis of 24hUPro control regarding combined immunosuppressants.

**FIGURE 12:**
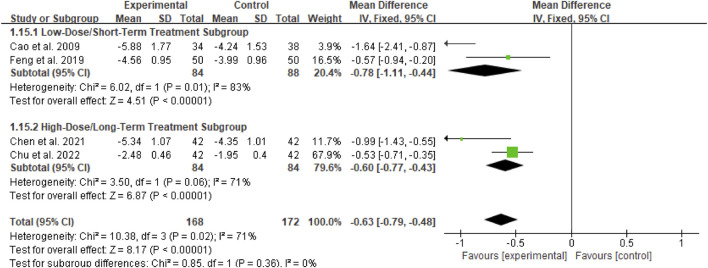
Subgroup analysis of 24hUPro control regarding dosage and treatment duration.

For the 24-h urinary protein (24Upro) outcome, subgroup analyses were conducted based on two dimensions: whether Bailing Capsule treatment for adult PNS was combined with immunosuppressive agents, and the dosage and treatment duration of Bailing Capsules. The results are shown in Figures 11, 12.

First, subgroup analysis was performed based on concurrent use of immunosuppressive agents (Figure 11):

Immunosuppressive Agent Combination Subgroup: This subgroup included 2 studies (Chen et al., 2021; Chu et al., 2022). The subgroup analysis showed that Bailing Capsules combined with immunosuppressive agents significantly reduced 24Upro levels (MD: −0.71; 95% CI: −1.16, −0.27; p = 0.002), with moderate between-study heterogeneity (I^2^ = 71%).

Non-Immunosuppressive Agent Subgroup: This subgroup included 2 studies, (Cao, 2009; Feng, 2019). The subgroup analysis showed that Bailing Capsules monotherapy also significantly reduced 24Upro levels (MD: −1.05; 95% CI: −2.09, −0.01; p = 0.05), with high between-study heterogeneity (I^2^ = 83%). Moreover, the test for subgroup differences revealed no statistically significant difference in the effects between the two subgroups (χ^2^ = 0.34, df = 1, p = 0.56; I^2^ = 0%).

Second, subgroup analysis was performed based on dosage and treatment duration (Figure 12):

Low-Dose/Short-Term Treatment Subgroup: This subgroup included 2 studies, (Cao, 2009; Feng, 2019). The subgroup analysis showed that low-dose short-term Bailing Capsule treatment significantly reduced 24Upro levels (MD: −0.78; 95% CI: −1.11, −0.44; p < 0.00001), with high between-study heterogeneity (I^2^ = 83%).

High-Dose/Long-Term Treatment Subgroup: This subgroup included 2 studies, (Chen and Wu, 2017; Chu et al., 2022). The subgroup analysis showed that high-dose long-term Bailing Capsule treatment also significantly reduced 24Upro levels (MD: −0.60; 95% CI: −0.77, −0.43; p < 0.00001), with moderate between-study heterogeneity (I^2^ = 71%). Furthermore, the test for subgroup differences indicated no statistically significant difference in the effects between the two subgroups (χ^2^ = 0.85, df = 1, p = 0.36; I^2^ = 0%).

##### 3.4.9.2 Subgroup analysis of ALB

**FIGURE 13:**
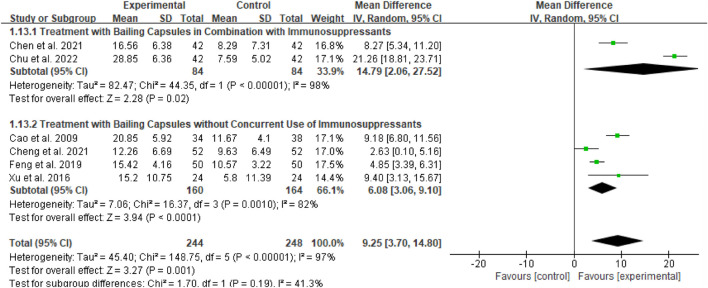
Subgroup analysis of ALB control regarding combined immunosuppressants.

**FIGURE 14:**
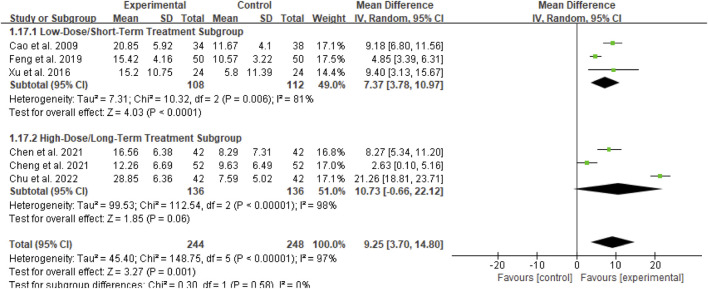
Subgroup analysis of ALB control regarding dosage and treatment duration.

For the ALB outcome, subgroup analyses were conducted based on two dimensions: whether Bailing Capsule treatment for adult PNS was combined with immunosuppressive agents, and the dosage and treatment duration of Bailing Capsules. The results are shown in Figures 13, 14.

First, subgroup analysis was performed based on concurrent use of immunosuppressive agents (Figure 13):

Immunosuppressive Agent Combination Subgroup: This subgroup included 2 studies (Chen et al., 2021; Chu et al., 2022). The subgroup analysis showed that Bailing Capsules combined with immunosuppressive agents significantly increased ALB levels (MD: 14.79; 95% CI: 2.06, 27.52; p = 0.02), with high between-study heterogeneity (I^2^ = 98%).

Non-Immunosuppressive Agent Subgroup: This subgroup included 4 studies (Cao, 2009; Cheng et al., 2021; Feng, 2019; Xu, 2016). The subgroup analysis showed that Bailing Capsules monotherapy also significantly increased ALB levels (MD: 6.08; 95% CI: 3.06, 9.10; p < 0.0001), with high between-study heterogeneity (I^2^ = 82%). Moreover, the test for subgroup differences revealed no statistically significant difference in the effects between the two subgroups (χ^2^ = 1.70, df = 1, p = 0.19; I^2^ = 41.3%).

Second, subgroup analysis was performed based on the dosage of Bailing Capsules (Figure 14):

Low-Dose/Short-Term Treatment Subgroup: This subgroup included 3 studies (Cao, 2009; Feng, 2019; Xu, 2016). The subgroup analysis showed that low-dose Bailing Capsule treatment significantly increased ALB levels (MD: 7.37; 95% CI: 3.78, 10.97; p < 0.0001), with high between-study heterogeneity (I^2^ = 81%).

High-Dose/Long-Term Treatment Subgroup: This subgroup included 3 studies (Chen et al., 2021; Cheng et al., 2021; Chu et al., 2022). The subgroup analysis showed that high-dose Bailing Capsule treatment had a marginal effect on increasing ALB levels (MD: 10.73; 95% CI: 0.66, 22.12; p = 0.06), with extremely high between-study heterogeneity (I^2^ = 98%). Furthermore, the test for subgroup differences indicated no statistically significant difference in the effects between the two subgroups (χ^2^ = 0.30, df = 1, p = 0.58; I^2^ = 0%).

##### 3.4.9.3 Subgroup analysis of BUN

**FIGURE 15:**
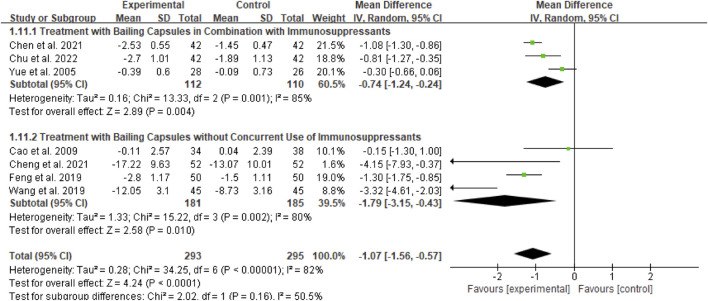
Subgroup analysis of BUN control regarding combined immunosuppressants.

**FIGURE 16:**
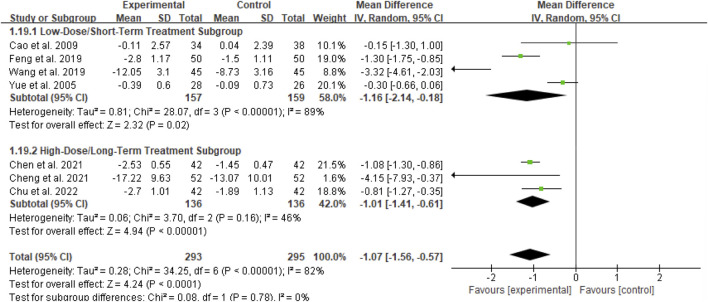
Subgroup analysis of BUN control regarding dosage and treatment duration.

For the BUN outcome, subgroup analyses were conducted based on two dimensions: whether Bailing Capsule treatment for adult PNS was combined with immunosuppressive agents, and the dosage and treatment duration of Bailing Capsules. The results are shown in Figures 15, 16.

First, subgroup analysis was performed based on concurrent use of immunosuppressive agents (Figure 15):

Immunosuppressive Agent Combination Subgroup: This subgroup included 3 studies (Yue et al., 2006; Chen et al., 2021; Chu et al., 2022). The subgroup analysis showed that Bailing Capsules combined with immunosuppressive agents significantly reduced BUN levels (MD: −0.74; 95% CI: −1.24, −0.24; p = 0.004), with high between-study heterogeneity (I^2^ = 85%).

Non-Immunosuppressive Agent Subgroup: This subgroup included 4 studies (Cao, 2009; Feng, 2019; Wang, 2019; Cheng et al., 2021). The subgroup analysis showed that Bailing Capsules monotherapy also significantly reduced BUN levels (MD: −1.79; 95% CI: −3.15, −0.43; p = 0.010), with high between-study heterogeneity (I^2^ = 80%). Moreover, the test for subgroup differences revealed no statistically significant difference in the effects between the two subgroups (χ^2^ = 2.02, df = 1, p = 0.16; I^2^ = 50.5%).

Second, subgroup analysis was performed based on dosage and treatment duration (Figure 16):

Low-Dose/Short-Term Treatment Subgroup: This subgroup included 4 studies (Yue et al., 2006; Cao, 2009; Feng, 2019; Wang, 2019). The subgroup analysis showed that low-dose/short-term Bailing Capsule treatment significantly reduced BUN levels (MD: −1.16; 95% CI: −2.14, −0.18; p = 0.02), with high between-study heterogeneity (I^2^ = 89%).

High-Dose/Long-Term Treatment Subgroup: This subgroup included 3 studies (Chen et al., 2021; Cheng et al., 2021; Chu et al., 2022). The subgroup analysis showed that high-dose/long-term Bailing Capsule treatment also significantly reduced BUN levels (MD: −1.01; 95% CI: −1.41, −0.61; p < 0.00001), with moderate between-study heterogeneity (I^2^ = 46%). Furthermore, the test for subgroup differences indicated no statistically significant difference in the effects between the two subgroups (χ^2^ = 0.08, df = 1, p = 0.78; I^2^ = 0%).

##### 3.4.9.4 Subgroup analysis of Scr

**FIGURE 17:**
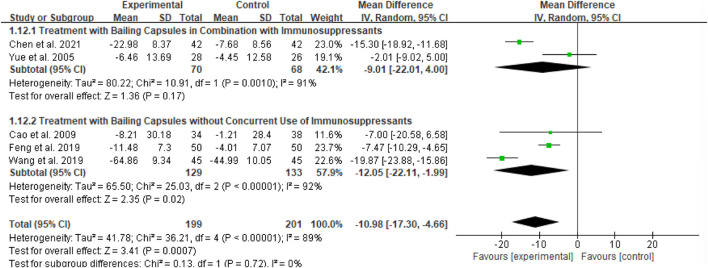
Subgroup analysis of Scr control regarding combined immunosuppressants.

**FIGURE 18:**
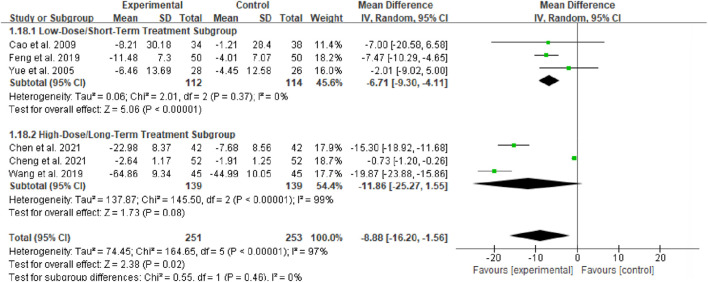
Subgroup analysis of Scr control regarding dosage and treatment duration.

For the Scr outcome, subgroup analyses were conducted based on two dimensions: whether Bailing Capsule treatment for adult PNS was combined with immunosuppressive agents, and the dosage and treatment duration of Bailing Capsules. The results are shown in Figures 17, 18.

First, subgroup analysis was performed based on concurrent use of immunosuppressive agents (Figure 17):

Immunosuppressive Agent Combination Subgroup: This subgroup included two studies (Yue et al., 2006; Chen et al., 2021). The subgroup analysis showed that Bailing Capsules combined with immunosuppressive agents had no statistically significant effect on reducing Scr levels (MD: −9.01; 95% CI: −22.01, 4.00; p = 0.17), with high between-study heterogeneity (I^2^ = 91%).

Non-Immunosuppressive Agent Subgroup: This subgroup included three studies (Cao, 2009; Feng, 2019; Wang, 2019). The subgroup analysis showed that Bailing Capsules monotherapy significantly reduced Scr levels (MD: −12.05; 95% CI: −22.11, −1.99; p = 0.02), with high between-study heterogeneity (I^2^ = 92%). Moreover, the test for subgroup differences revealed no statistically significant difference in the effects between the two subgroups (χ^2^ = 0.13, df = 1, p = 0.72; I^2^ = 0%).

Second, subgroup analysis was performed based on dosage and treatment duration (Figure 18):

Low-Dose/Short-Term Treatment Subgroup: This subgroup included three studies (Yue et al., 2005; Cao, 2009; Feng, 2019). The subgroup analysis showed that low-dose/short-term Bailing Capsule treatment significantly reduced Scr levels (MD: −6.71; 95% CI: −9.30, −4.11; p < 0.00001), with no between-study heterogeneity (I^2^ = 0%).

High-Dose/Long-Term Treatment Subgroup: This subgroup included three studies (Wang, 2019; Chen et al., 2021; Cheng et al., 2021). The subgroup analysis showed that high-dose/long-term Bailing Capsule treatment had a marginal effect on reducing Scr levels (MD: −11.86; 95% CI: −25.27, 1.55; p = 0.08), with extremely high between-study heterogeneity (I^2^ = 99%). Furthermore, the test for subgroup differences indicated no statistically significant difference in the effects between the two subgroups (χ^2^ = 0.55, df = 1, p = 0.46; I^2^ = 0%).

### 3.5 Quality of evidence assessment

The evidence quality for the incidence of adverse events, 24hUpro, ALB, TC, Scr, BUN, CD3^+^, CD4^+^, and the CD4^+^/CD8^+^ was rated as low, primarily due to substantial inconsistency (high between-study heterogeneity) and/or imprecision (a limited number of included studies and relatively small sample sizes for some outcomes). In contrast, the evidence quality for clinical efficacy rate and adverse event rate was downgraded to a lesser extent and was therefore rated as moderate (Table 3).

**TABLE 3:** Quality of evidence assessment.

Outcome and follow-up	Patients (studies), N	Relative effect (95% CI)	Absolute effects (95% CI)			Certainty
			Control group	Bailing capsule	Difference	
Efficient	770 (9 RCTs)	**RR = 1.22** (1.15–1.29)	775 per 1,000	**946 per 1,000** (1,000–891)	**171 more per 1,000** (from 116 more to 225 more)	⊕⊕⊕○ Moderate^a^
ALB	493 (6 RCTs)	—	0	—	**9.25** (3.7–14.8)	⊕⊕○○ Low^a^ ^,^ ^b^
24Upro	340 (4 RCTs)	—	0	—	**−0.81** (−1.17 to −0.45)	⊕⊕○○ Low^a^ ^,^ ^c^
BUN	588 (7 RCTs)	—	0	—	**−1.07** (−1.56 to −0.57)	⊕⊕○○ Low^a^ ^,^ ^b^
TC	175 (2 RCTs)	—	0	—	**−1.62** (−1.98 to −1.25)	⊕⊕○○ Low^a^ ^,^ ^c^
Scr	400 (5 RCTs)	—	0	—	**−7.37** (−20.23 to 5.48)	⊕○○○ Very low^a^ ^,b,^ ^c^ ^,^ ^d^
CD3^+^	198 (3 RCTs)	—	0	—	**22.96** (−20.53–66.44)	⊕○○○ Very low^a^ ^,b,^ ^c^ ^,^ ^d^
CD4^+^	198 (3 RCTs)	—	0	—	**6.55** (4.18–8.91)	⊕○○○ Very low^a^ ^,^ ^c^ ^,^ ^b^
CD4^+^/CD8^+^	198 (3 RCTs)	—	0	—	**0.34** (0.26–0.43)	⊕⊕○○ Low^a^ ^,^ ^b^
Adverse reaction rate	444 (5 RCTs)	**RR = 0.55** (0.32–0.93)	156 per 1,000	**86 per 1,000** (145–50)	**70 fewer per 1,000** (from 106 fewer to 11 fewer)	⊕⊕⊕○ Moderate^a^
**CI:** Confidence interval; **MD:** mean difference; **RR:** risk ratio						

^a^ Lowered by one level: None of the studies mentioned the implementation of blinding and allocation concealment.

^b^ Lowered by one level:Heterogeneity test I2 greater than 50%.

^c^ Lowered by one level: Studies with fewer than five included.

^d^ Lowered by one level: The confidence interval crosses the clinical decision threshold.

Bold values indicate statistically significant results: for risk ratios (RR), bold values represent 95% confidence intervals not crossing 1; for mean differences (MD), bold values represent 95% confidence intervals not crossing 0 (P < 0.05).

### 3.6 Assessment of publication bias

**FIGURE 19:**
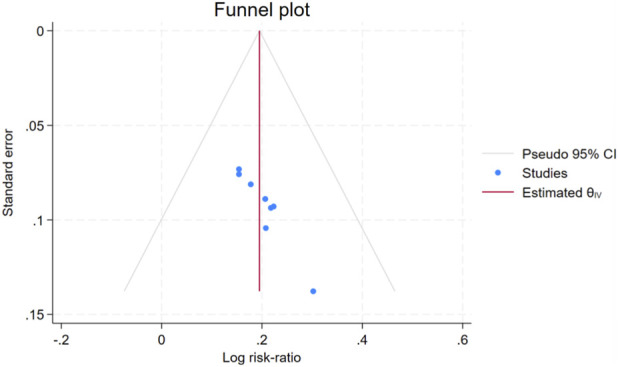
Funnel plot of efficacy rate.

Visual inspection of funnel plots for the clinical efficacy rate suggested that the included studies were generally symmetrically distributed around the pooled effect size (log risk ratio). Although a small number of studies showed slight asymmetry, the overall distribution appeared largely symmetrical (Figure 19). Furthermore, Egger’s test for the clinical efficacy rate indicated a P value of 0.2465 (>0.05), suggesting no significant publication bias for this outcome.

## 4 Discussion

Based on the included RCTs, this meta-analysis demonstrates that Bailing Capsule, whether used as monotherapy or as an adjunct to conventional therapy, confers significant clinical benefits for adult patients with PNS. Specifically, treatment with Bailing Capsule was associated with significant reductions in 24hUpro, Scr, BUN, and TC, alongside marked increases in ALB, CD3^+^, CD4^+^, and the CD4^+^/CD8^+^. Moreover, compared with conventional therapy alone, patients receiving Bailing Capsule achieved a higher overall clinical response rate and a lower incidence of adverse events, suggesting a favorable benefit–risk profile.

Reduction of proteinuria and correction of hypoalbuminemia are central therapeutic goals in PNS, as persistent proteinuria and low ALB levels are closely associated with disease progression and adverse renal outcomes. The present findings suggest that Bailing Capsule significantly decreases 24hUpro while increasing ALB levels, suggesting that it may alleviate edema and promote disease remission by improving glomerular filtration barrier integrity and enhancing protein synthesis or reducing protein loss. In addition, the observed reductions in Scr and BUN imply a renoprotective effect, potentially contributing to delayed deterioration of renal function in adult patients with PNS.

### 4.1 Potential mechanisms of action of Bailing Capsule

Bailing Capsule is a standardized fungal medicinal preparation officially listed in the *Chinese Pharmacopoeia* (2020 Edition). Its renoprotective effects are thought to arise from the synergistic actions of multiple bioactive metabolites—such as cordyceps polysaccharides, cordycepic acid (mannitol), adenosine, and cordycepin—through multi-target and multi-pathway mechanisms.

- Protection of the glomerular filtration barrier and tubular function. Cordyceps polysaccharides can inhibit mesangial cell proliferation and excessive extracellular matrix (ECM) deposition, attenuate podocyte injury, and thereby reduce proteinuria. Adenosine modulates glomerular endothelial function, improves renal microcirculation, and promotes repair of tubular epithelial cells. Hirsutella sinensis mycelia have been shown to suppress autophagy-related AMPK/ULK1 signaling, alleviating D-galactose–induced tubular epithelial cell senescence and reducing LC3 expression, thus delaying tubular functional decline (Liu et al., 2019). In addition, nucleoside and nucleobase metabolites of *Ophiocordyceps sinensis* (CS-N) inhibit epithelial–mesenchymal transition (EMT), downregulate α-SMA, fibronectin, and collagen I, and reduce ECM accumulation via inhibition of the p38/ERK MAPK pathway, thereby attenuating renal fibrosis (Dong et al., 2019).
- Bidirectional immunomodulation. As key immunoregulatory metabolites, cordyceps polysaccharides activate macrophages, T and B lymphocytes, and natural killer cells. Through pathways such as TLR4/MyD88/p38 and NF-κB, they increase CD3^+^ and CD4^+^ levels and the CD4^+^/CD8^+^, and promote the secretion of cytokines including IL-2, IL-12, and IFN-γ. These effects help correct immune dysfunction while suppressing aberrant immune responses and immune complex deposition. Furthermore, cordyceps polysaccharides enhance local IgA secretion, improve mucosal immune deficiency, reverse tumor-associated macrophage M2 polarization, inhibit PD-L1/PD-1–mediated T-cell suppression, and regulate the Treg/Th2 balance (Chen et al., 2024; Das et al., 2021).
- Anti-inflammatory and oxidative stress regulation. By inhibiting NF-κB signaling, Bailing Capsule reduces the release of pro-inflammatory mediators such as TNF-α, IL-6, and hs-CRP, while upregulating the anti-inflammatory cytokine IL-10 and restoring Th1/Th2 balance (Liang et al., 2022). Cordycepic acid (mannitol) and polysaccharides enhance the activities of superoxide dismutase (SOD) and glutathione peroxidase (GPx), reduce malondialdehyde (MDA) levels, and mitigate oxidative stress–induced renal cellular injury (Liu et al., 2019; Das et al., 2021). Cordycepin suppresses ferroptosis via GSK-3β–mediated activation of Nrf2, thereby reducing lipid peroxidation in renal tissues (Tang et al., 2024), and inhibits NF-κB–mediated Gasdermin D cleavage, attenuating macrophage pyroptosis and inflammatory infiltration in the kidney (Tang and Zhu, 2024; Dong et al., 2019).
- Improvement of renal hemodynamics and metabolic regulation. Bailing Capsule promotes nitric oxide (NO) production through modulation of nitric oxide synthase, inhibits activation of the renin–angiotensin–aldosterone system (RAAS), protects vascular endothelial function, and reduces blood pressure elevation during renal ischemia–reperfusion injury. Through PI3K/Akt and HIF-1 signaling pathways, it improves renal microcirculation (Liang et al., 2022; Wang et al., 2013; Yuan et al., 2013). Moreover, its nucleoside and nucleobase metabolites reduce Scr, BUN, and urinary albumin levels in diabetic nephropathy models and ameliorate disturbances in glucose and lipid metabolism, while cordycepin alleviates hyperuricemia-related renal injury by regulating URAT1 expression (Du et al., 2019; Liu et al., 2022).
- Anti-fibrotic and cytoprotective effects. When combined with immunosuppressive or anticoagulant therapies, Bailing Capsule may attenuate tubulointerstitial fibrosis by inhibiting the TGF-β and p38/ERK MAPK pathways, reduce thrombotic risk, and preserve renal blood perfusion. Cordyceps polysaccharides suppress high-glucose–induced EMT in tubular epithelial cells, reduce collagen deposition, and delay the progression of diabetic nephropathy, while enhancing antioxidant enzyme expression to protect renal parenchymal cells from apoptosis (Dong et al., 2019; Liang et al., 2022; Das et al., 2021).

Overall, these pleiotropic biological effects provide mechanistic support for the observed clinical benefits of Bailing Capsule in adult PNS and underscore its potential role as an effective adjunctive therapeutic option.

### 4.2 Comparison with previous studies

The findings of the present study are broadly consistent with those of previous systematic reviews and meta-analyses investigating Bailing Capsule or *O. sinensis*–based preparations for the treatment of chronic kidney disease or nephrotic syndrome (Xu et al., 2020; Tao et al., 2024; Zhou et al., 2024). However, in contrast to earlier reports, the current analysis not only evaluated clinical response rates, safety outcomes, proteinuria reduction, and renal function improvement, but also incorporated a wider range of outcome measures, including TC, ALB, and indices of T-lymphocyte–mediated immune function.

Moreover, several prior meta-analyses included heterogeneous disease entities or pooled adult and pediatric populations. By contrast, the present study focused exclusively on adult patients with PNS, thereby providing more targeted and clinically relevant evidence to inform therapeutic decision-making in this specific population.

### 4.3 Sensitivity analysis and publication bias

Sensitivity analyses demonstrated that the beneficial effects of Bailing Capsule on key renal and metabolic outcomes were generally robust. Nevertheless, relatively high heterogeneity was observed for several endpoints, including BUN, Scr, ALB, and 24hUpro. One potential contributor to this heterogeneity may be differences in concomitant use of immunosuppressive agents.

Subgroup analyses stratified by concomitant immunosuppressive therapy showed that, with the exception of Scr, both subgroups consistently favored Bailing Capsule for improvements in 24hUpro, ALB, and BUN, with no statistically significant differences between subgroups (all P > 0.05). These findings suggest that the use of immunosuppressants is unlikely to represent the primary source of heterogeneity and further support the stability of the renoprotective effects of Bailing Capsule in adult PNS, irrespective of background immunosuppressive therapy.

The lack of statistical significance for Scr reduction in the subgroup receiving concomitant immunosuppressants (P = 0.17) may be attributable to the limited number of included studies (n = 2) and small sample size (n = 70). Consequently, this finding should be interpreted with caution and warrants confirmation in larger, well-designed trials with standardized treatment protocols.

Another potential contributor to heterogeneity was investigated through subgroup analyses stratified by the dosage and treatment duration of Bailing Capsule (low-dose/short-term vs. high-dose/long-term). For Scr, the low-dose/short-term subgroup demonstrated complete elimination of between-study heterogeneity (I^2^ = 0%), while substantial heterogeneity persisted in the high-dose/long-term subgroup (I^2^ = 99%). Notably, the test for subgroup differences revealed no statistically significant discrepancy in effect sizes between the two dosage-duration subgroups (P = 0.46), yet the stark contrast in heterogeneity levels suggests that dosage and treatment duration may represent a key driver of Scr-specific heterogeneity, likely driven by greater variability in baseline renal function and longer follow-up periods in high-dose/long-term studies. For 24hUpro, ALB, and BUN, subgroup analyses by dosage and duration showed consistent beneficial effects of Bailing Capsule in both subgroups, with no statistically significant differences between subgroups (all P > 0.05), and heterogeneity remained largely unexplained. These results indicate that while dosage and treatment duration partially account for Scr heterogeneity, other unmeasured factors (e.g., pathological subtypes of PNS, baseline disease severity, or concomitant renoprotective medications) may contribute to the residual heterogeneity across most outcomes.

Although subgroup analyses did not fully eliminate heterogeneity, they clarified the overall consistency of treatment effects across different therapeutic contexts, thereby providing supportive evidence for the use of Bailing Capsule in combination with diverse treatment regimens. Importantly, sensitivity analyses indicated that removal of any single study did not materially alter the pooled effect estimates, underscoring the robustness of the overall conclusions.

With respect to publication bias, Egger’s test for overall clinical response rates yielded a P value >0.05, and funnel plots for the primary outcomes appeared largely symmetrical. Nonetheless, the possibility of publication bias cannot be entirely excluded, as studies reporting negative or null results may be underrepresented due to lower likelihood of publication, potentially leading to a modest overestimation of treatment effects.

### 4.4 Safety analysis

None of the included studies reported serious adverse events attributable to Bailing Capsule. Only a small proportion of patients experienced mild gastrointestinal discomfort (e.g., abdominal distension or diarrhea) or transient pharyngeal discomfort, which generally resolved spontaneously after administration with meals or dose adjustment and did not necessitate treatment discontinuation. These findings suggest that Bailing Capsule is generally well tolerated. However, evidence regarding its long-term safety remains limited, highlighting the need for high-quality clinical trials with extended follow-up durations.

### 4.5 Strengths and limitations of the study

This meta-analysis has several notable strengths. First, a relatively comprehensive outcome framework was adopted. To our knowledge, this is the first study to simultaneously evaluate clinical response rate, incidence of adverse events, 24hUpro, ALB, Scr, TC, BUN, and immunological parameters including CD3^+^, CD4^+^, and the CD4^+^/CD8^+^, thereby enabling a multidimensional assessment of the overall therapeutic profile of Bailing Capsule. Second, the included trials encompassed a variety of combination regimens, allowing clarification of its potential clinical utility across different therapeutic contexts and providing supportive evidence for individualized treatment strategies. Third, this review was conducted in strict accordance with the PRISMA statement, with methodological rigor ensured through application of the Cochrane risk-of-bias tool and the GRADE framework for evidence quality assessment, while sensitivity analyses were performed to further confirm the robustness of the pooled results.

Several limitations should also be acknowledged. First, all included studies were conducted in Chinese populations, resulting in limited ethnic diversity and warranting caution when extrapolating the findings to other populations. Second, grey literature (e.g., unpublished trial data or academic theses) was not searched, which may have led to omission of potentially relevant studies. Third, all outcome indicators selected in this study were short-term efficacy measures, and long-term prognostic indicators such as the incidence of end-stage renal disease (ESRD), time to renal function deterioration, and incidence of cardiovascular complications were not included, which may restrict the comprehensive assessment of the long-term clinical value of Bailing Capsule. Forth, Subgroup analysis based on pathological subtypes (MCD/FSGS/MN) was not performed due to insufficient data in original studies. Furthermore, the present study only performed a meta-analysis on TC, while quantitative analyses of triglyceride (TG), low-density lipoprotein cholesterol (LDL-C), and high-density lipoprotein cholesterol (HDL-C) could not be conducted. The main reason was that among the two included studies, one only reported post-treatment data without baseline values, which failed to meet the methodological requirements for a valid meta-analysis. This represents an additional limitation of this study.Finally, owing to the limited number of available studies, comparisons among different combination regimens could not be performed, nor could an optimal dose–response relationship for Bailing Capsule in adult PNS be determined, highlighting the need for further investigation.

## 5 Conclusion

In summary, this systematic review and meta-analysis suggests that Bailing Capsule, used either as an adjunct to or an alternative for conventional therapy, may confer beneficial effects in adult patients with PNS by reducing proteinuria, improving plasma albumin levels, preserving renal function, ameliorating dyslipidemia, modulating immune function, and enhancing overall clinical efficacy, with a favorable safety profile. Despite these encouraging findings, the limitations related to ethnic homogeneity, relatively small sample sizes for certain outcomes, and the lack of comparative analyses across different combination strategies underscore the need for future large-scale, rigorously designed, multicenter RCTs. Such studies should include ethnically diverse populations, directly compare various combination regimens with respect to cost-effectiveness, and incorporate longer follow-up periods to validate long-term renoprotective effects and the potential role of Bailing Capsule in delaying renal fibrosis progression.

## Data Availability

The original contributions presented in the study are included in the article/Supplementary Material, further inquiries can be directed to the corresponding authors.

## Glossary

T: treatment group
C: control group
Age: presented as mean ± standard deviation; range in parentheses Interventions
BC: Bailing Capsule
Tx: Treatment
Atorva: Atorvastatin
Pred: Prednisone
CTX: Cyclophosphamide
CsA: Cyclosporine A
UK: Urokinase
Pred Acetate: Prednisone Acetate
LMWH: Low Molecular Weight Heparin Outcomes
ALB: Albumin
24hPr: 24 h Urine Protein Quantitative
BUN: Blood Urea Nitrogen
TC: Total Cholesterol
PT: Prothrombin Time
Scr: Serum Creatinine
UNAG: Urine N-acetyl-β-D-glucosaminidase
11-DH-TXB2: 11-Dehydrothromboxane B2
HMGB-1: High Mobility Group Protein B1
hs-CRP: Hypersensitive C-reactive Protein
TNF-α: Tumor Necrosis Factor-α
IL: Interleukin
FIB: Fibrinogen
APTT: Activated Partial Thromboplastin Time
24hUTP: 24 h Urine Protein Quantitative
GFR: Glomerular Filtration Rate
KIM-1: Kidney Injury Molecule-1
RBP: Retinol Binding Protein
NAG: N-acetyl-β-D-glucosaminidase
24hUpro: 24 h Urine Protein
Plt: Platelet
ApoA1: Apolipoprotein A1
ApoB: Apolipoprotein B
β2-MG: β2-Microglobulin
LPa: Lipoprotein a
suPAR: Soluble Urokinase-type Plasminogen Activator Receptor
