# Supplementary figures and images for: Efficacy and safety of Bailing Capsule for the treatment of adult primary nephrotic syndrome: a systematic review and meta-analysis of randomized controlled trials
Source: Front Pharmacol. 2026 Apr 24;17:1798042. doi: 10.3389/fphar.2026.1798042 (PMC13153111; doi:10.3389/fphar.2026.1798042)

Figure S1:

Figure S2


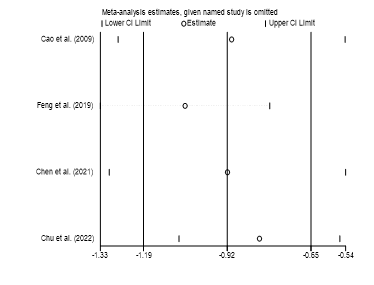


Figure S3

Figure S4


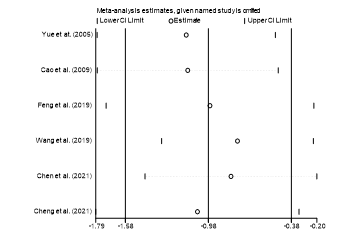


Figure S5a


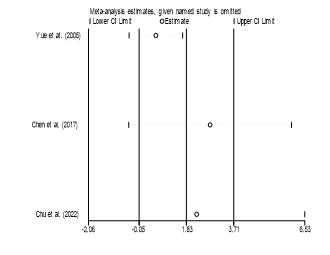


Figure S5b


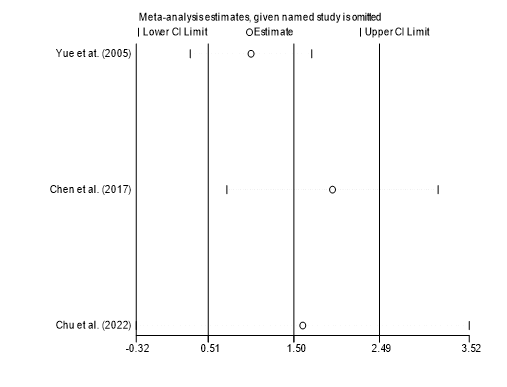


Figure S5c


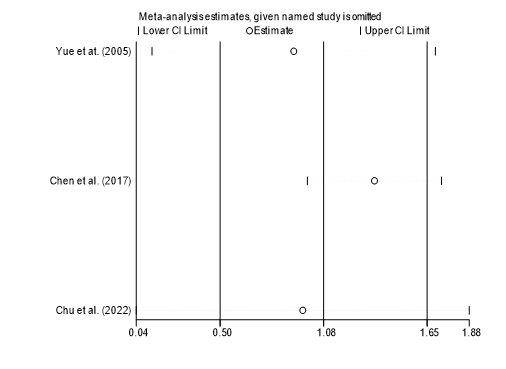


Figure S6


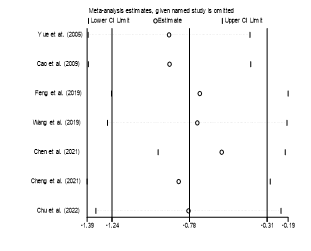


Figure S7


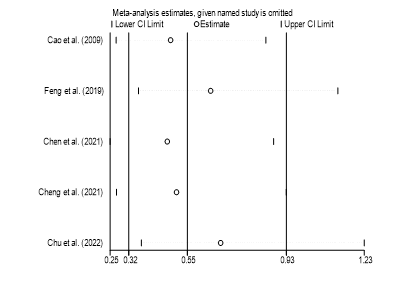

Supplement: Supplementary file 2 [file Supplementaryfile2.doc]
