# Supplementary material for: Efficacy and safety of Bailing Capsule for the treatment of adult primary nephrotic syndrome: a systematic review and meta-analysis of randomized controlled trials
Source: Front Pharmacol. 2026 Apr 24;17:1798042. doi: 10.3389/fphar.2026.1798042 (PMC13153111; doi:10.3389/fphar.2026.1798042)
Supplement: Supplementary file 3 [file Supplementaryfile1.docx]

Efficacy and Safety of Bailin Capsules in the Treatment of Nephrotic Syndrome: A Systematic Review and Meta-Analysis of Randomized Controlled Trials

Search formula：

CNKI：134 articles

（主题：百令胶囊 + 中成药百令胶囊 + 百令 + 冬虫夏草 + 冬虫夏草菌粉 + 冬虫夏草制剂 + 发酵冬虫夏草菌粉 + Bailing）

AND

（主题：肾病综合征 + 肾病综合症 + 肾综 + 膜性肾病 + 微小病变 + 局灶节段性肾小球硬化 + FSGS + nephrotic syndrome + NS）

WangFang：162 articles

（主题：百令胶囊 OR 中成药百令胶囊 OR 百令 OR 冬虫夏草 OR 冬虫夏草菌粉 OR 冬虫夏草制剂 OR 发酵冬虫夏草菌粉 OR Bailing）

AND

（主题：肾病综合征 OR 肾病综合症 OR 肾综 OR 膜性肾病 OR 微小病变 OR 局灶节段性肾小球硬化 OR FSGS OR nephrotic syndrome OR NS）

VIP：90 articles

（题名或关键词：百令胶囊 OR 中成药百令胶囊 OR 百令 OR 冬虫夏草 OR 冬虫夏草菌粉 OR 冬虫夏草制剂 OR 发酵冬虫夏草菌粉 OR Bailing）

AND

（题名或关键词：肾病综合征 OR 肾病综合症 OR 肾综 OR 膜性肾病 OR 微小病变 OR 局灶节段性肾小球硬化 OR FSGS OR nephrotic syndrome OR NS）

Sinomed：166 articles

( "百令胶囊"[常用字段:智能] OR "中成药百令胶囊"[常用字段:智能] OR "百令"[常用字段:智能] OR "冬虫夏草"[常用字段:智能] OR "冬虫夏草菌粉"[常用字段:智能] OR "冬虫夏草制剂"[常用字段:智能] OR "发酵冬虫夏草菌粉"[常用字段:智能] OR "Bailing"[常用字段:智能]) AND

( "肾病综合征"[常用字段:智能] OR "肾病综合症"[常用字段:智能] OR "肾综"[常用字段:智能] OR "膜性肾病"[常用字段:智能] OR "微小病变"[常用字段:智能] OR "局灶节段性肾小球硬化"[常用字段:智能] OR "FSGS"[常用字段:智能] OR "nephrotic syndrome"[常用字段:智能] OR "NS"[常用字段:智能])

Pubmed：1 articles

Bailing capsule OR Bailing OR Ophiocordyceps sinensis OR Cordyceps sinensis OR Sphaeria sinensis OR Caterpillar Fungus OR Fungus, Caterpillar OR Cordyceps militaris

Nephrotic Syndrome OR Nephrotic Syndromes OR Syndrome, Nephrotic OR Steroid-Dependent Nephrotic Syndrome OR Nephrotic Syndrome, Steroid-Dependent OR Steroid Dependent Nephrotic Syndrome OR Steroid-Dependent Nephrotic Syndromes OR Steroid-Resistant Nephrotic Syndrome OR Nephrotic Syndrome, Steroid-Resistant OR Steroid Resistant Nephrotic Syndrome OR Steroid-Resistant Nephrotic Syndromes OR Childhood Idiopathic Nephrotic Syndrome OR Pediatric Idiopathic Nephrotic Syndrome OR Frequently Relapsing Nephrotic Syndrome OR Steroid-Sensitive Nephrotic Syndrome OR Nephrotic Syndrome, Steroid-Sensitive OR Steroid Sensitive Nephrotic Syndrome OR Steroid-Sensitive Nephrotic Syndromes OR Syndrome, Steroid-Sensitive Nephrotic OR Multi-Drug Resistant Nephrotic Syndrome OR Multi Drug Resistant Nephrotic Syndrome

(Bailing capsule[Title/Abstract] OR Bailing[Title/Abstract] OR Ophiocordyceps sinensis[Title/Abstract] OR Cordyceps sinensis[Title/Abstract] OR Sphaeria sinensis[Title/Abstract] OR Caterpillar Fungus[Title/Abstract] OR Fungus, Caterpillar[Title/Abstract] OR Cordyceps militaris[Title/Abstract])

AND

(Nephrotic Syndrome[MeSH] OR Nephrotic Syndromes[Title/Abstract] OR Syndrome, Nephrotic[Title/Abstract] OR Steroid-Dependent Nephrotic Syndrome[Title/Abstract] OR Nephrotic Syndrome, Steroid-Dependent[Title/Abstract] OR Steroid Dependent Nephrotic Syndrome[Title/Abstract] OR Steroid-Dependent Nephrotic Syndromes[Title/Abstract] OR Steroid-Resistant Nephrotic Syndrome[Title/Abstract] OR Nephrotic Syndrome, Steroid-Resistant[Title/Abstract] OR Steroid Resistant Nephrotic Syndrome[Title/Abstract] OR Steroid-Resistant Nephrotic Syndromes[Title/Abstract] OR Childhood Idiopathic Nephrotic Syndrome[Title/Abstract] OR Pediatric Idiopathic Nephrotic Syndrome[Title/Abstract] OR Frequently Relapsing Nephrotic Syndrome[Title/Abstract] OR Steroid-Sensitive Nephrotic Syndrome[Title/Abstract] OR Nephrotic Syndrome, Steroid-Sensitive[Title/Abstract] OR Steroid Sensitive Nephrotic Syndrome[Title/Abstract] OR Steroid-Sensitive Nephrotic Syndromes[Title/Abstract] OR Syndrome, Steroid-Sensitive Nephrotic[Title/Abstract] OR Multi-Drug Resistant Nephrotic Syndrome[Title/Abstract] OR Multi Drug Resistant Nephrotic Syndrome[Title/Abstract])

Web of science：4 articles

Bailing capsule OR Bailing OR Ophiocordyceps sinensis OR Cordyceps sinensis OR sphaerica sinensis OR Caterpillar Fungus OR Fungus, Caterpillar OR Cordyceps militaris (Topic)

and

Nephrotic Syndrome OR Nephrotic Syndromes OR Syndrome, Nephrotic OR Steroid-Dependent Nephrotic Syndrome OR Nephrotic Syndrome, Steroid-Dependent OR Steroid Dependent Nephrotic Syndrome OR Steroid-Dependent Nephrotic Syndromes OR Steroid-Resistant Nephrotic Syndrome OR Nephrotic Syndrome, Steroid-Resistant OR Steroid Resistant Nephrotic Syndrome OR Steroid-Resistant Nephrotic Syndromes OR Childhood Idiopathic Nephrotic Syndrome OR Pediatric Idiopathic Nephrotic Syndrome OR Frequently Relapsing Nephrotic Syndrome OR Steroid-Sensitive Nephrotic Syndrome OR Nephrotic Syndrome, Steroid-Sensitive OR Steroid Sensitive Nephrotic Syndrome OR Steroid-Sensitive Nephrotic Syndromes OR Syndrome, Steroid-Sensitive Nephrotic OR Multi-Drug Resistant Nephrotic Syndrome OR Multi Drug Resistant Nephrotic Syndrome (Topic)

Embase：4 articles

('bailing capsule':ti,ab,kw OR bailing:ti,ab,kw OR 'ophiocordyceps sinensis':ti,ab,kw OR 'cordyceps sinensis':ti,ab,kw OR 'sphaerica sinensis':ti,ab,kw OR 'caterpillar fungus':ti,ab,kw OR 'fungus, caterpillar':ti,ab,kw OR 'cordyceps militaris':ti,ab,kw) AND ('nephrotic syndrome':ti,ab,kw OR 'nephrotic syndromes':ti,ab,kw OR 'syndrome, nephrotic':ti,ab,kw OR 'steroid-dependent nephrotic syndrome':ti,ab,kw OR 'nephrotic syndrome, steroid-dependent':ti,ab,kw OR 'steroid dependent nephrotic syndrome':ti,ab,kw OR 'steroid-dependent nephrotic syndromes':ti,ab,kw OR 'steroid-resistant nephrotic syndrome':ti,ab,kw OR 'nephrotic syndrome, steroid-resistant':ti,ab,kw OR 'steroid resistant nephrotic syndrome':ti,ab,kw OR 'steroid-resistant nephrotic syndromes':ti,ab,kw OR 'childhood idiopathic nephrotic syndrome':ti,ab,kw OR 'pediatric idiopathic nephrotic syndrome':ti,ab,kw OR 'frequently relapsing nephrotic syndrome':ti,ab,kw OR 'steroid-sensitive nephrotic syndrome':ti,ab,kw OR 'nephrotic syndrome, steroid-sensitive':ti,ab,kw OR 'steroid sensitive nephrotic syndrome':ti,ab,kw OR 'steroid-sensitive nephrotic syndromes':ti,ab,kw OR 'syndrome, steroid-sensitive nephrotic':ti,ab,kw OR 'multi-drug resistant nephrotic syndrome':ti,ab,kw OR 'multi drug resistant nephrotic syndrome':ti,ab,kw)

Cochrane：0 articles

Bailing capsule OR Bailing OR Caterpillar Fungus OR Ophiocordyceps sinensis OR Sphaeria sinensis OR Fungus, Caterpillar OR Cordyceps sinensis OR Cordyceps militaris

Nephrotic Syndrome, Steroid-Resistant OR Steroid-Resistant Nephrotic Syndrome OR Steroid-Resistant Nephrotic Syndromes OR Steroid Resistant Nephrotic Syndrome OR Multi Drug Resistant Nephrotic Syndrome OR Multi-Drug Resistant Nephrotic Syndrome OR Nephrotic Syndrome, Steroid-Dependent OR Steroid Dependent Nephrotic Syndrome OR Steroid-Dependent Nephrotic Syndromes OR Steroid-Dependent Nephrotic Syndrome OR Frequently Relapsing Nephrotic Syndrome OR Steroid-Sensitive Nephrotic Syndromes OR Nephrotic Syndrome, Steroid-Sensitive OR Steroid-Sensitive Nephrotic Syndrome OR Syndrome, Steroid-Sensitive Nephrotic OR Steroid Sensitive Nephrotic Syndrome OR Pediatric Idiopathic Nephrotic Syndrome OR Childhood Idiopathic Nephrotic Syndrome OR Nephrotic Syndromes OR Syndrome, Nephrotic

Bailing capsule OR Bailing OR Caterpillar Fungus OR Ophiocordyceps sinensis OR Sphaeria sinensis OR Fungus, Caterpillar OR Cordyceps sinensis OR Cordyceps militaris in Title Abstract Keyword

AND

Nephrotic Syndrome, Steroid-Resistant OR Steroid-Resistant Nephrotic Syndrome OR Steroid-Resistant Nephrotic Syndromes OR Steroid Resistant Nephrotic Syndrome OR Multi Drug Resistant Nephrotic Syndrome OR Multi-Drug Resistant Nephrotic Syndrome OR Nephrotic Syndrome, Steroid-Dependent OR Steroid Dependent Nephrotic Syndrome OR Steroid-Dependent Nephrotic Syndromes OR Steroid-Dependent Nephrotic Syndrome OR Frequently Relapsing Nephrotic Syndrome OR Steroid-Sensitive Nephrotic Syndromes OR Nephrotic Syndrome, Steroid-Sensitive OR Steroid-Sensitive Nephrotic Syndrome OR Syndrome, Steroid-Sensitive Nephrotic OR Steroid Sensitive Nephrotic Syndrome OR Pediatric Idiopathic Nephrotic Syndrome OR Childhood Idiopathic Nephrotic Syndrome OR Nephrotic Syndromes OR Syndrome, Nephrotic in Title Abstract Keyword
